# Supplementary material for: A combination of two human monoclonal antibodies cures symptomatic rabies
Source: EMBO Mol Med. 2020 Sep 18;12(11):e12628. doi: 10.15252/emmm.202012628 (PMC7645379; doi:10.15252/emmm.202012628)
Supplement: Supplementary file 1 — Appendix [file EMMM-12-e12628-s001.pdf]

A combination of two human monoclonal antibodies cures symptomatic rabies

Guilherme Dias de Melo[1§], Florian Sonthonnax[1,2§], Gabriel Lepousez[3], Grégory Jouvion[4,5], Andrea Minola[6], Fabrizia Zatta[6], Florence Larrous[1], Lauriane Kergoat[1], Camille Mazo[3], Carine Moigneu[3], Roberta Aiello[7], Angela Salomoni[7], Elise Brisebard[4,8], Paola De Benedictis[7], Davide Corti[6] and Hervé Bourhy[1*]

Appendix

Table of contents

Figures

- Appendix Figure S1. PK profile of RVC20 and RVC58 monoclonal antibodies in mice.

- Appendix Figure S2. Cytokines and innate immune mediators' profile in the brain of Tha-RABV infected mice at the different time points chosen to start the mAbs treatment.

- Appendix Figure S3. Viral load, cytokines and innate immune mediators' profile in the brain of Tha-RABV infected mice and treated by intramuscular and intracerebroventricular administration of the RVC20-LALA and RVC58-LALA monoclonal antibodies cocktail.

Tables

- Appendix Table S1. Kinetics of brain invasion by Tha-RABV and microglial reaction in infected mice at the different time points chosen to start the mAbs treatment.

- Appendix Table S2. Exact P-values and statistical tests.

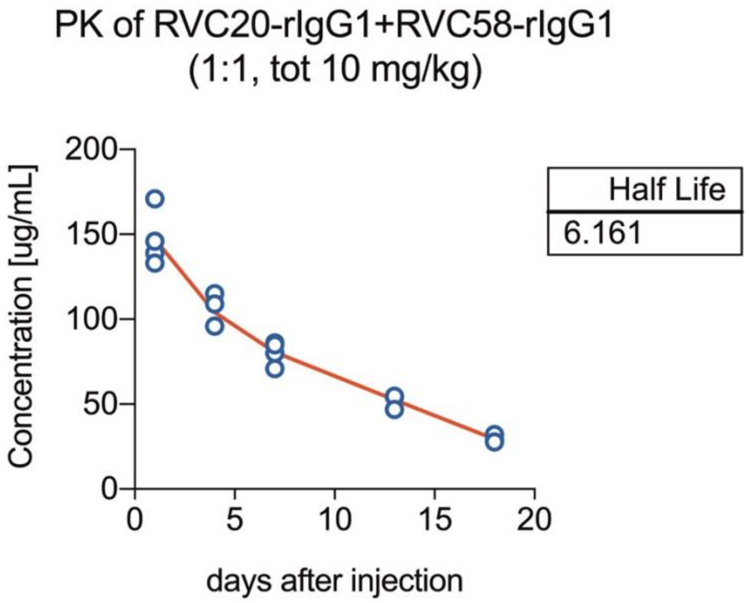

Appendix Figure S1. PK profile of RVC20 and RVC58 monoclonal antibodies in mice. A mix of RVC20-rIgG1 (5 mg/kg) and RVC58-rIgG1 (5 mg/kg) was injected intravenously in B6/HuFcRn mice (n=4), the serum concentration of this antibody cocktail was measured at 1, 4, 7, 13 and 18 days post-injection and the half-life was determined using GraphPad Prism.

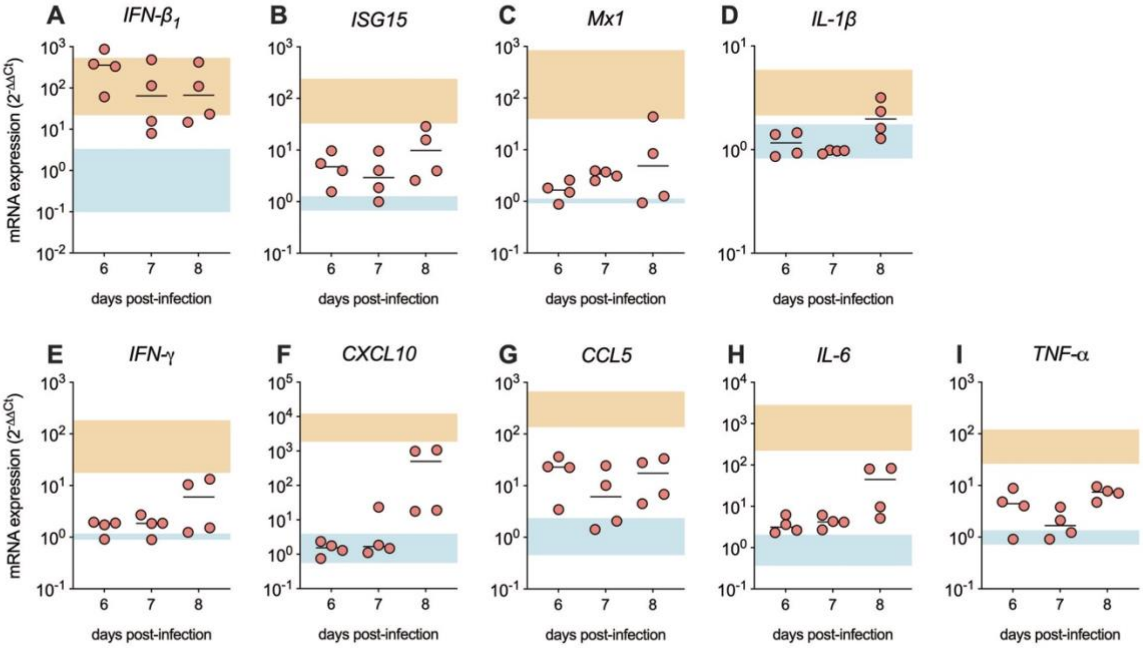

Appendix Figure S2. Cytokines and innate immune mediators' profile in the brain of Tha-RABV infected mice at the different time points chosen to start the mAbs treatment. Gene expression of (**A**) IFN-β_1, (**B**) ISG15, (**C**) Mx1, (**D**) IL-1β, (**E**) IFN-γ, (**F**) CXCL10, (**G**) CCL5, (**H**) IL-6, and (**I**) TNF-α in one brain hemisphere of mice at 6, 7 and 8 days post-infection (n=4 per time-point). The expression of the genes of interest was normalized to the GAPDH housekeeping gene. Horizontal lines indicate the median. The orange crosshatched areas correspond to the 95%CI of the median from the infected and non-treated mice, and the blue crosshatched areas correspond to the 95%CI of the median from the non-infected mice (see Figure 2).

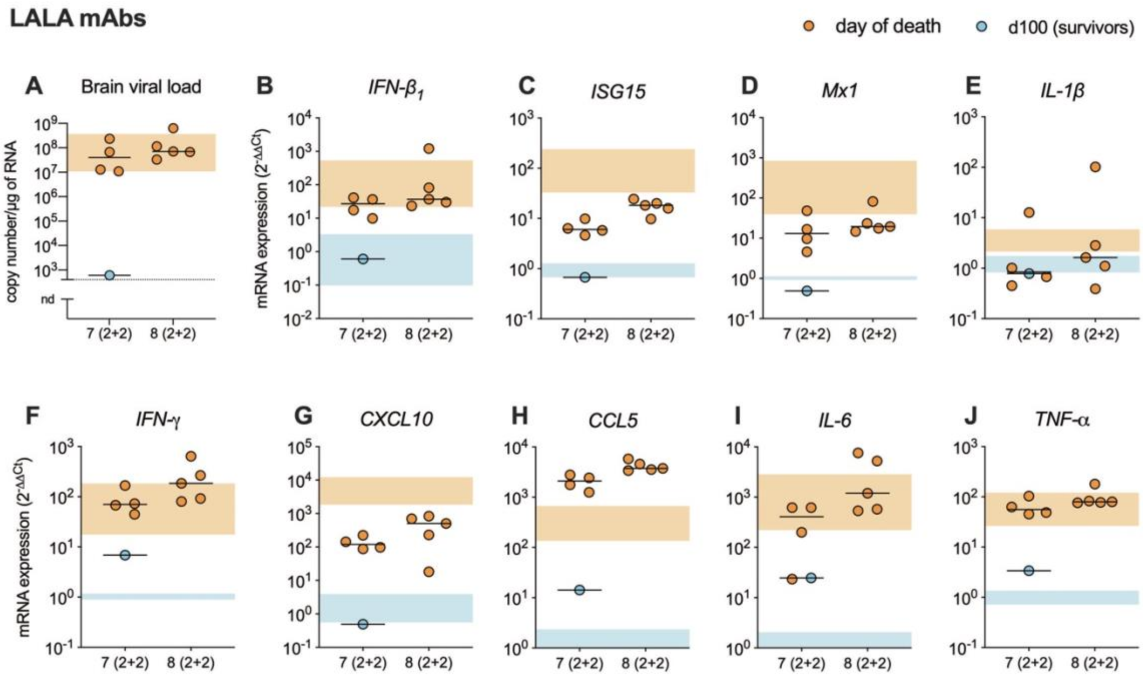

Appendix Figure S3. Viral load, cytokines and innate immune mediators' profile in the brain of Tha-RABV infected mice and treated by intramuscular and intracerebroventricular administration of the RVC20-LALA and RVC58-LALA monoclonal antibodies cocktail. (**A**) Viral load in the brain of mice from different experimental groups. The samples were either collected at the time of death, or at 100 dpi for the survivors. Gene expression of (**B**) IFN-β1, (**C**) ISG15, (**D**) Mx1, (**E**) IL-1β, (**F**) IFN-γ, (**G**) CXCL10, (**H**) CCL5, (**I**) IL-6, and (**J**) TNF-α in one brain hemisphere of the infected and treated mice. The expression of the genes of interest was normalized to the GAPDH housekeeping gene. Horizontal lines indicate the median. The orange crosshatched areas correspond to the 95%CI of the median from the infected and non-treated mice, and the blue crosshatched areas correspond to the 95%CI of the median from the non-infected mice (see Figure 2). Treatments description= 7 (2+2): infected, treated at 7 dpi (2+2 mg/kg) (n=5); 8 (2+2): infected, treated at 8 dpi (2+2 mg/kg) (n=5).

Appendix Table S1. Kinetics of brain invasion by Tha-RABV and microglial reaction in infected mice at the different time points chosen to start the mAbs treatment.

days post-infection	Brain area	Viral distribution (Tha-RABV)		Microglial cell reaction (Iba1+ cells)	
		% of animals (n=4)	intensity	% of animals (n=4)	intensity
6 dpi	Medulla oblongata/Pons	-	-	-	-
	Midbrain	-	-	-	-
	Cerebellum	-	-	-	-
	Hypothalamus	-	-	-	-
	Thalamus	-	-	-	-
	Cortex	-	-	-	-
7 dpi	Medulla oblongata/Pons	25%	±	-	-
	Midbrain	-	-	-	-
	Cerebellum	-	-	-	-
	Hypothalamus	-	-	-	-
	Thalamus	-	-	-	-
	Cortex	-	-	-	-
8 dpi	Medulla oblongata/Pons	75%	± to ++	50%	+ to ++
	Midbrain	50%	± to +++	75%	±
	Cerebellum	25%	+	25%	±
	Hypothalamus	50%	± to ++	25%	++
	Thalamus	25%	+	-	-
	Cortex	25%	+	25%	±

immunohistochemical semi-quantitative analysis:
- absent | ± discrete | + mild | ++ moderate | +++ severe

Appendix Table S2. Exact P-values and statistical tests.

Figure	Compared groups	P-value	Test
Fig 1E	7 days post-infection	0.0029	Kruskal-Wallis followed by the Dunn' multiple comparisons test
	8 days post-infection	0.0015	Kruskal-Wallis followed by the Dunn' multiple comparisons test
	20 days post-infection, non-infected vs. infected, treated at 8 dpi (2+2 mg/kg)	0.0167	Mann-Whitney test
	30 days post-infection, non-infected vs. infected, treated at 8 dpi (2+2 mg/kg)	0.0167	Mann-Whitney test
	60 days post-infection, non-infected vs. infected, treated at 8 dpi (2+2 mg/kg)	0.0167	Mann-Whitney test
Fig 2A	infected, non-treated vs. infected, treated at 6 dpi (2+2 mg/kg)	0.0062	Log-rank (Mantel-Cox) test
	infected, non-treated vs. infected, treated at 7 dpi (2+2 mg/kg)	0.0001	Log-rank (Mantel-Cox) test
	infected, non-treated vs. infected, treated at 8 dpi (2+2 mg/kg)	0.0002	Log-rank (Mantel-Cox) test
	infected, non-treated vs. infected, treated at 8 dpi (10+10 mg/kg)	0.0062	Log-rank (Mantel-Cox) test
Fig 2B	infected, non-treated vs. infected, treated	<0.0001	Log-rank (Mantel-Cox) test
Fig EV1A	nt vs. ni	0.0027	Log-rank (Mantel-Cox) test
Fig EV1B	nt vs. 2 dpi	0.0027	Log-rank (Mantel-Cox) test
	nt vs. 4 dpi	0.0027	Log-rank (Mantel-Cox) test
	nt vs. 6 dpi	0.0027	Log-rank (Mantel-Cox) test
Fig EV1C	nt vs. 2 dpi	0.0027	Log-rank (Mantel-Cox) test
	nt vs. 4 dpi	0.0027	Log-rank (Mantel-Cox) test
	nt vs. 6 dpi	0.0027	Log-rank (Mantel-Cox) test
	nt vs. 8 dpi	0.0143	Log-rank (Mantel-Cox) test
Fig EV2A	infected, non-treated vs. infected, treated at 2 dpi	<0.0001	Log-rank (Mantel-Cox) test
	infected, non-treated vs. infected, treated at 3 dpi	<0.0001	Log-rank (Mantel-Cox) test
Fig EV3E	infected, non-treated vs. infected, treated at 7 dpi	0.0084	Log-rank (Mantel-Cox) test
	infected, non-treated vs. infected, treated at 8 dpi	0.0203	Log-rank (Mantel-Cox) test
