# Supplementary material for: A combination of two human monoclonal antibodies cures symptomatic rabies
Source: EMBO Mol Med. 2020 Sep 18;12(11):e12628. doi: 10.15252/emmm.202012628 (PMC7645379; doi:10.15252/emmm.202012628)
Supplement: Supplementary file 2 — Expanded View Figures PDF [file EMMM-12-e12628-s002.pdf]

Expanded View Figures

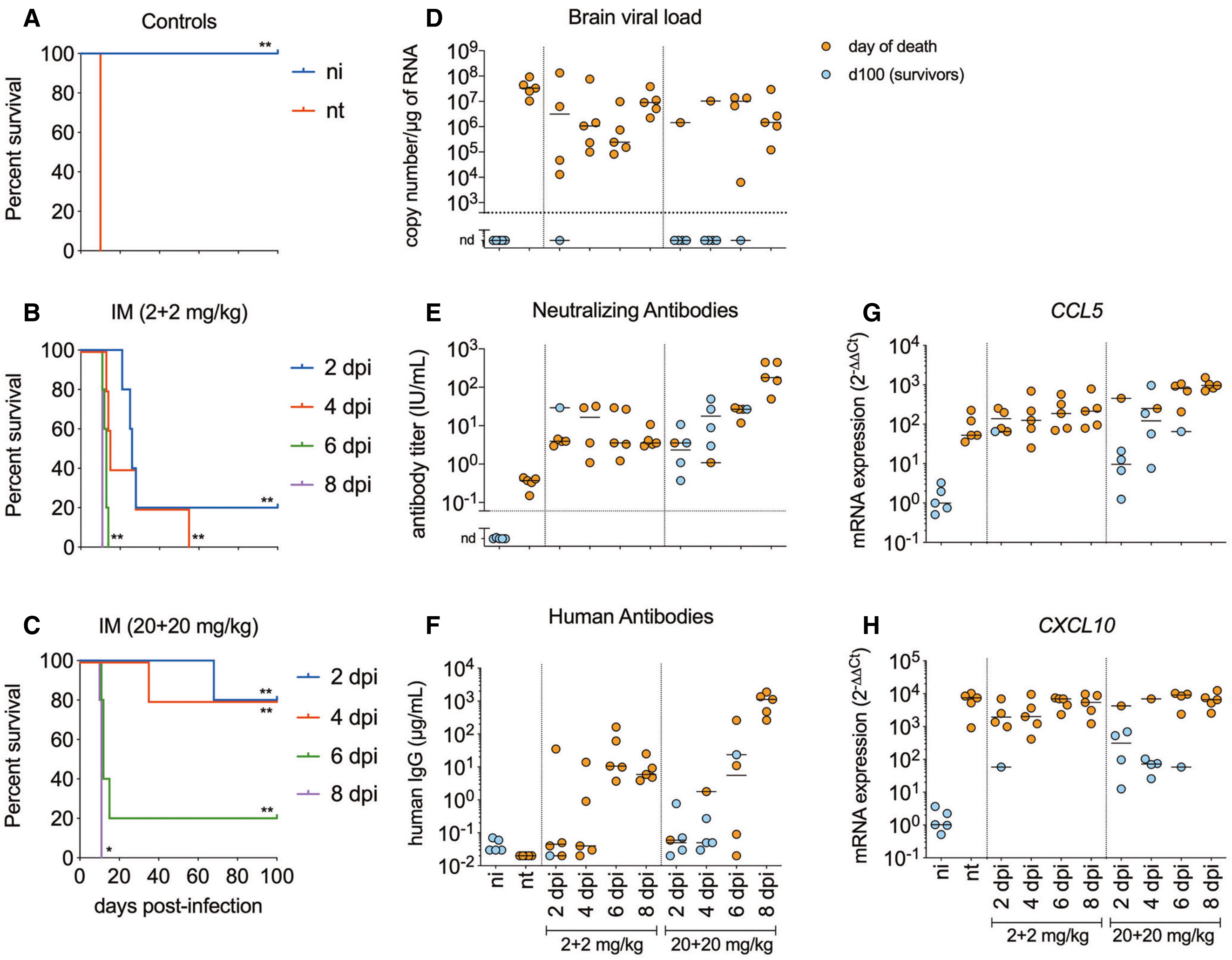

Figure EV1. Efficacy of intramuscular administration of the RVC20 and RVC58 monoclonal antibody cocktail in Tha-RABV-infected mice.

A–C Kaplan–Meier survival curve of non-treated mice (A), or treated at different time points with a single intramuscular injection of 1:1 combination of RVC20 and RVC58 human monoclonal antibodies at the dose of 2 + 2 mg/kg (B) and 20 + 20 mg/kg (C) (n = 5 per treatment). Of note, the survivor treated at 6 dpi presented with persistent monoplegia as sequelae of the infection. Statistical analysis was performed using log-rank (Mantel–Cox) test with $\alpha = 0.05$. *$P < 0.05$, **$P < 0.01$. ni: non-infected; nt: infected, non-treated. Exact P values are shown in Appendix Table S2.

D–H Brain viral load (D) detected in the brain, virus-neutralizing antibodies (e), and human antibodies (F) detected in the serum of mice from different experimental groups, and the relative brain expression of *CCL5* (G) and *CXCL10* (H). Data information: Horizontal lines indicate the median. The expression of the genes of interest was normalized to the GAPDH housekeeping gene. The samples were either collected at the time of death, or at 100 dpi for the survivors. ni: non-infected; nt: infected, non-treated; nd: not detected (n = 5 per group).

© 2020 The Authors

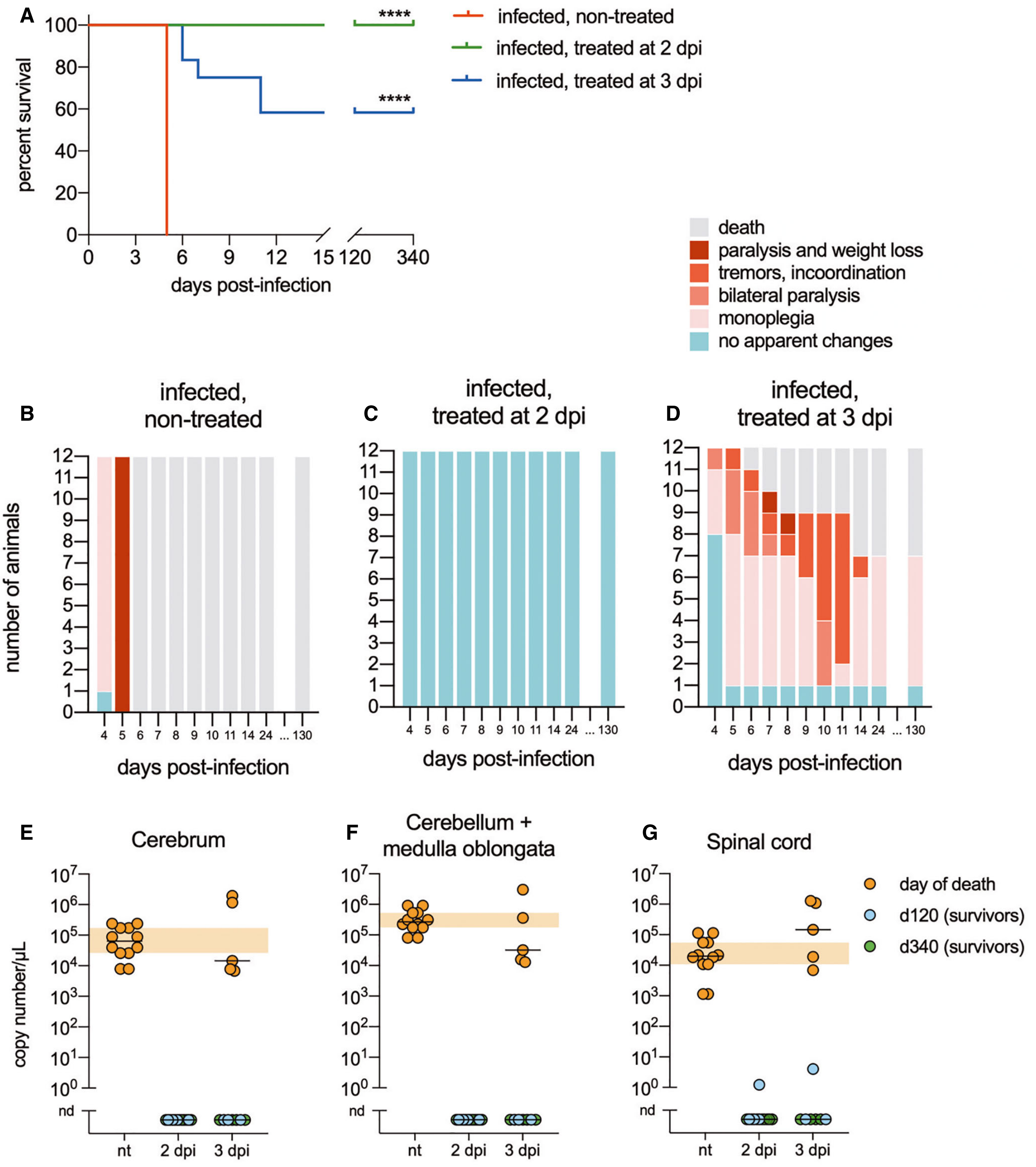

Figure EV2.

 © 2020 The Authors

Figure EV2. Efficacy of intramuscular administration of the RVC20 and RVC58 monoclonal antibody cocktail in CVS-11-infected golden Syrian hamsters.

A Cumulative Kaplan–Meier survival curves of hamsters under different treatments (*n* = 12 per treatment). Log-rank (Mantel–Cox) test to compare treated groups with the infected, non-treated group. ****$P < 0.0001$.

B–D Follow-up of the clinical signs of hamsters under different treatments, based on a progressive 0–5 clinical score scale.

E–G Viral load in the cerebrum (E), cerebellum and medulla oblongata (F), and spinal cord (G) of hamsters from different experimental groups. The samples were either collected at the time of death, or at 120 and 340 dpi for the survivors. Data information: Horizontal lines indicate the median. The orange crosshatched areas correspond to the 95% CI of the median from the infected and non-treated hamsters. nt: infected, non-treated; nd: not detected (*n* = 12 per group).

Figure EV3. Intracerebroventricular (ICV) drug delivery in mice, and the therapeutic efficacy of intramuscular and ICV administration of the RVC20-LALA and RVC58-LALA antibody cocktail against rabies.

A Stereotaxic implantation of brain infusion kit connected to iPRECIO pump in mice to deliver the monoclonal antibody cocktail in the right lateral ventricle. The stereotaxic coordinates, taken bregma as reference, were −0.5 mm anteroposterior, +1.0 mm mediolateral (arrow), and −2.4 mm dorsoventral.

B, C Non-infected mice received fluorescent antibodies (goat IgG anti-chicken IgY conjugated to Alexa Fluor® 647; Invitrogen A-21449) by ICV, 10 μg/day during 3 days (1, *n* = 1), by intraperitoneal injection, 10 μg/mouse, 6 h before imaging (2, *n* = 1), and non-injected control (3, *n* = 1). Mice were euthanized, and the brains were extracted and imaged using the IVIS Spectrum (PerkinElmer); the fluorescence in the brain was detected only in the animal receiving the antibodies by ICV, with a diffusion spectrum from the site of injection throughout the brain (B; top panels: dorsal view of the whole brain; bottom panels: sagittal views). The total fluorescence was quantified in the whole brain (C).

D Neutralization of Tha-RABV by RVC20 and RVC58 antibodies, wild type (WT), or LALA, alone or in 1:1 combination. Data are expressed as IC50 (ng/ml). Horizontal lines indicate mean ± SD for two independent experiments.

E–G Therapeutic efficacy of intramuscular and intracerebroventricular administration of the RVC20-LALA and RVC58-LALA antibody cocktail against rabies. (E) Kaplan–Meier survival curves of mice under different treatments (*n* = 5 per treatment). Log-rank (Mantel–Cox) test: *$P < 0.05$, **$P < 0.01$. (F–G) Follow-up of the clinical signs (F) and body weight (G) of mice under different treatments. Heat maps were established based on a progressive 0–7 clinical score scale (0: no apparent changes; 1: ruffled fur; 2: slow movement, hind limb ataxia; 3: apathy; 4: monoplegia; 5: hind limb paralysis, tremors; 6: paralysis, conjunctivitis/keratitis, urine staining of the haircoat of the perineum; 7: death). Each line represents one animal throughout time. The infected, non-treated group is also displayed in Fig 2.

© 2020 The Authors

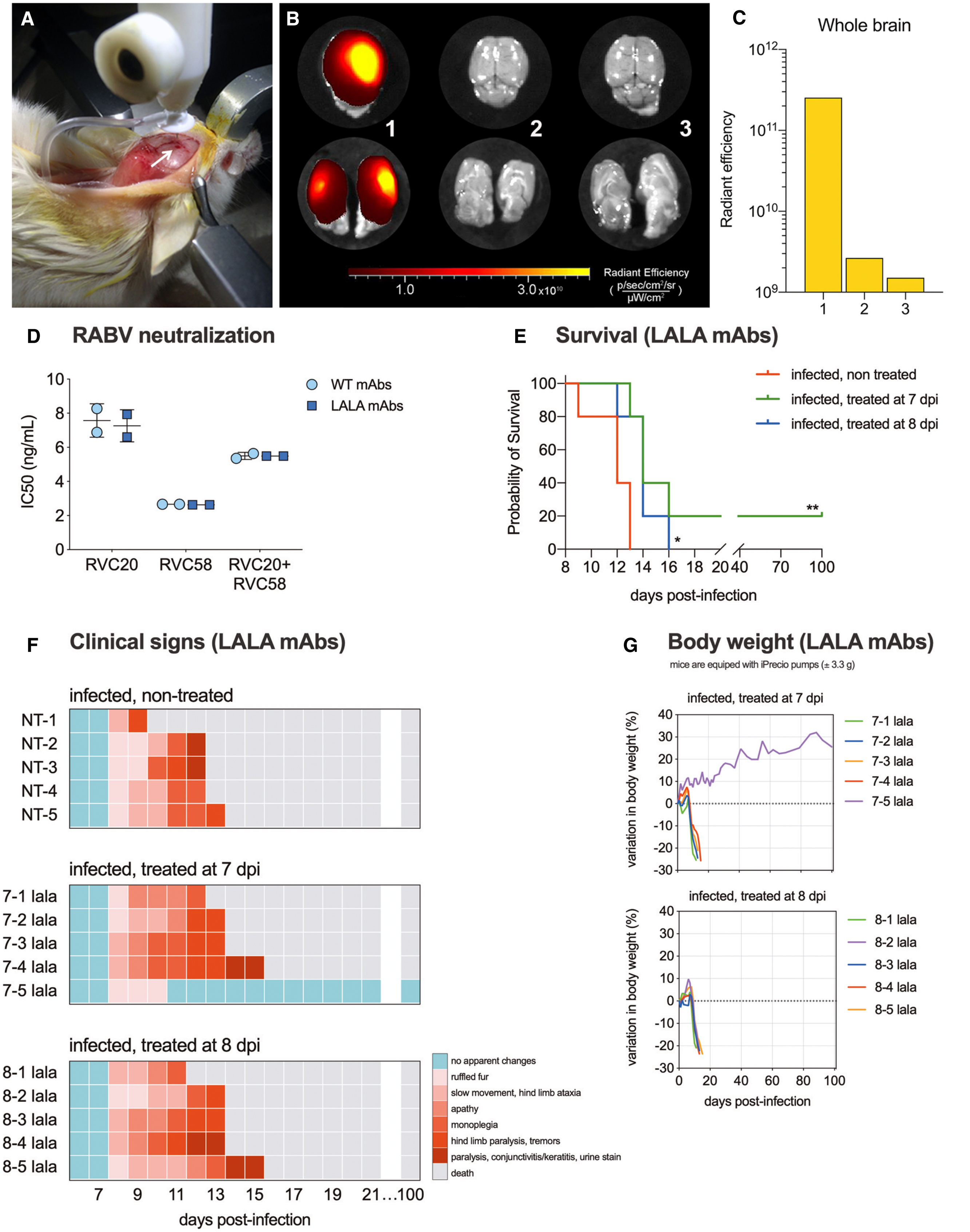

Figure EV3.

© 2020 The Authors

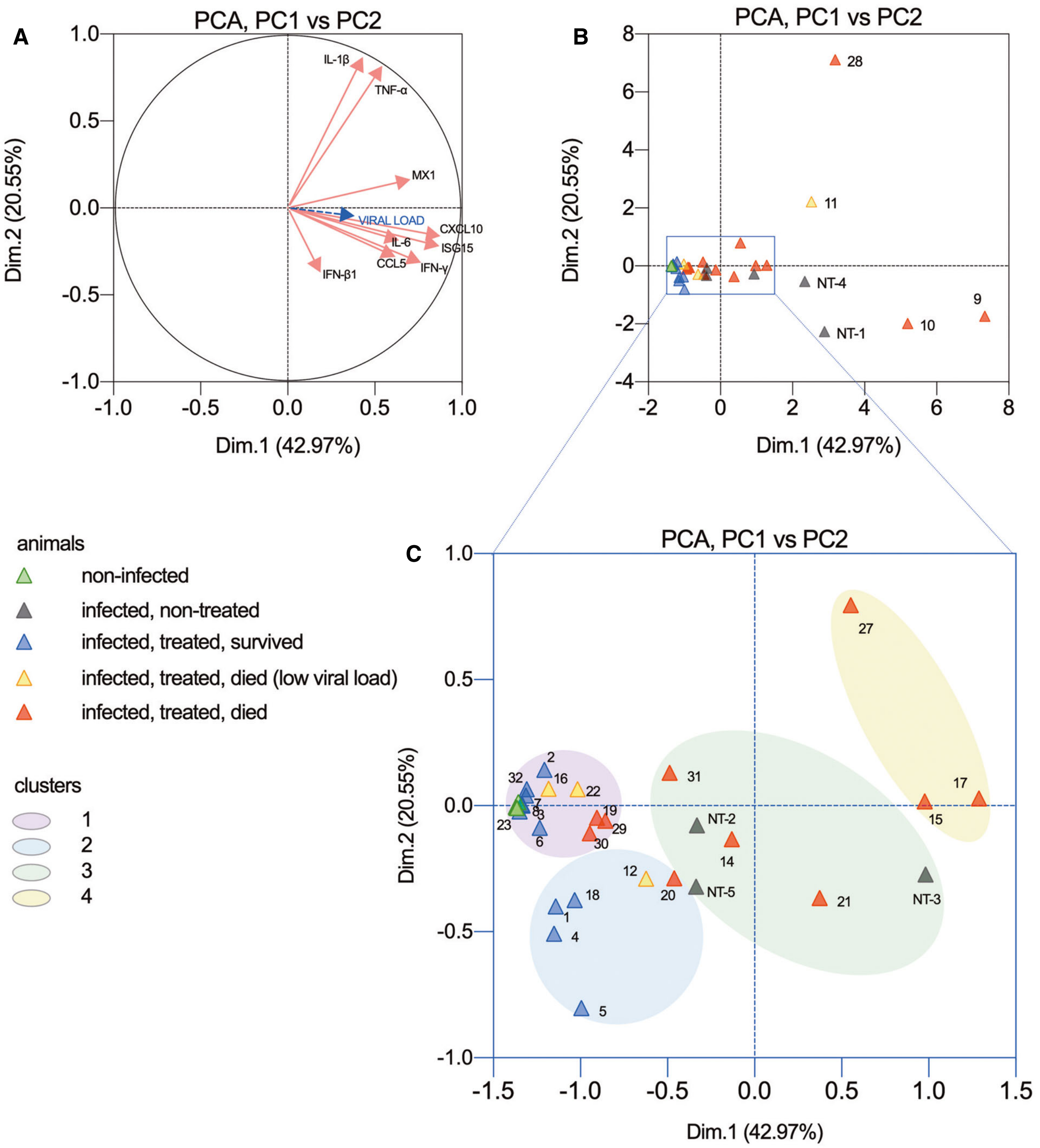

Figure EV4. Principal component analysis (PCA) of immune mediators in the brain of Tha-RABV-infected mice treated with intramuscular and intracerebroventricular administration of the RVC20 and RVC58 monoclonal antibody cocktail.

A Variable correlation plot showing the correlation of the gene expression of nine immune mediators in the brain of mice. The two-first principal components explained 63.65% of samples variability. *IFN-γ*, *CXCL10*, and the type I IFN-stimulated genes *ISG15* and *Mx1* contributed to PC1 (principal component 1; 66.7%), whereas *IL-1β* and *TNF-α* loaded positively on PC2 (principal component 2; 76.1%).

B, C PCA plots. Each symbol represents one animal, colored according to the experimental groups. Non-infected mice clustered together with survivors and even with some treated mice that died before the end of treatment. Four out of ten identified clusters are shown in the plot (C).

© 2020 The Authors
